# Serotonin System Implication in l-DOPA-Induced Dyskinesia: From Animal Models to Clinical Investigations

**DOI:** 10.3389/fneur.2014.00078

**Published:** 2014-05-20

**Authors:** Manolo Carta, Elisabetta Tronci

**Affiliations:** ^1^Section of Physiology, Department of Biomedical Sciences, University of Cagliari, Monserrato, Italy

**Keywords:** serotonin, dopamine, l-DOPA, dyskinesia, 5-HT1 receptor, Parkinson’s disease

## Abstract

In the recent years, the serotonin system has emerged as a key player in the induction of l-DOPA-induced dyskinesia (LID) in animal models of Parkinson’s disease. In fact, serotonin neurons possess the enzymatic machinery able to convert exogenous l-DOPA to dopamine (DA), and mediate its vesicular storage and release. However, serotonin neurons lack a feedback control mechanism able to regulate synaptic DA levels. While in a situation of partial DA depletion spared DA terminals can buffer DA released from serotonin neurons, the progression of DA neuron degeneration impairs this protective mechanism, causing swings in synaptic DA levels and pulsatile stimulation of post-synaptic DA receptors. In line with this view, removal of serotonin neurons by selective toxin, or pharmacological silencing of their activity, produced complete suppression of LID in animal models of Parkinson’s disease. In this article, we will revise the experimental evidence pointing to the important role of serotonin neurons in dyskinesia, and we will discuss the clinical implications.

## The Role of Striatal Pre-Synaptic Neurons in the Appearance of LID

l-DOPA, the precursor of dopamine (DA), represents the most effective drug for alleviating motor symptoms in Parkinson’s disease (PD) patients. Its efficacy is due to the ability to restore synaptic DA levels, an effect thought to be mediated by the spared dopaminergic neurons. Indeed, it is known that at the time of diagnosis something between 50 and 70% of nigral DA neurons have already degenerated; the remaining neurons, however, have the ability to take up the exogenous l-DOPA, convert it to DA, store DA into vesicles, and mediate its synaptic release (1, 2). The presence of the DA D2 auto-receptor at the pre-synaptic membrane, which activates a feedback control mechanism able to fine-tune the neurotransmitter release, allows the maintenance of physiological-like synaptic DA levels. Thus, preservation of this mechanism of regulation of DA release in spared DA terminals avoids excessive post-synaptic DA receptor stimulation following chronic l-DOPA administration in PD patients. Unfortunately, the so-called *honeymoon period* does not last forever; in fact, it is estimated that about 40 and 90% of patients suffer for motor fluctuations, known as l-DOPA-induced dyskinesia (LID), within the first 5 and 10 years from diagnosis, respectively. Thus, sooner or later, most of the patients experience a significant worsening of the therapeutic effect of l-DOPA and of their quality of life.

The current view on the risk factors underlying the development of dyskinesias suggests that the progression of the dopaminergic degeneration, rather than the duration of l-DOPA treatment, makes the therapeutic effect to deteriorate over-time (3). Accordingly, it has been shown that delaying the initiation of the l-DOPA administration (using direct DA receptor agonists for the first few years), while postpones the onset of dyskinesia compared to l-DOPA monotherapy, does not reduce the severity of dyskinesia once l-DOPA is introduced (4).

Interestingly, in a positron emission tomography (PET) study, it has been demonstrated that dyskinetic patients present higher synaptic DA levels 1 h after administration of l-DOPA compared to stable responders (5). Thus, dyskinesias appear to be associated to the inability to maintain synaptic DA levels within certain limits, which is likely caused by the progression of DA neuron degeneration and consequent reduced ability to mediate controlled DA release. In fact, in parkinsonian animals, severe dyskinesias develop only in subjects with extensive DA lesion, while partial lesioned animals show either none, or only mild dyskinesias. Elegant support to this view was provided by Ulusoy and collaborators (6); in this study, a significant DA deficiency was induced in rats by viral vector delivery of short hairpin RNA for the tyrosine hydroxylase (TH) enzyme. Interestingly, DA-depleted rats were completely resistant to the induction of dyskinesias following administration of a high dose of l-DOPA (even after being primed with apomorphine), opposite to control animals with similar striatal DA depletion. These results can be explained by the fact that striatal DA terminals were largely preserved after inhibition of DA synthesis, providing a buffering system for the exogenous administered l-DOPA. In this situation, synaptic levels of DA can be maintained within a physiological range by the presence of D2 auto-receptors and DA transporter (DAT) on striatal DA terminals. The ability of the pre-synaptic DA compartment to prevent excessive DA receptor stimulation, even in presence of supersensitive striatal DA receptors, is also confirmed in rat transplantation studies. In fact, l-DOPA-primed dyskinetic rats tend to normalize their response to l-DOPA after receiving ventral mesencephalic DA grafts into the lesioned striatum, which reconstitute the pre-synaptic buffering capacity. The reduction of LID is achieved despite post-synaptic DA receptors remain supersensitive, as shown by the abnormal dyskinetic response of these animals to amphetamine administration (7, 8).

As further support pointing to similar mechanisms underlying the appearance of dyskinesia in patients and animal models, dyskinetic rats have been shown to present higher extracellular DA levels compared to non-dyskinetic animals after administration of l-DOPA, as measured in microdialysis experiments (9, 10), similar to what seen by de la Fuente-Fernandez et al. (5) in dyskinetic patients, in the aforementioned PET-imaging study.

Due to the intermittent oral administration of l-DOPA, synaptic DA peaks are rapidly followed by minimal neurotransmitter levels, resulting in continuous fluctuations of synaptic DA concentration; this determines a pulsatile stimulation of post-synaptic striatal DA receptors which is considered to be the driving force for the induction of post-synaptic alterations at the level of striatal neurons (5, 11).

In agreement with the key detrimental role of synaptic DA swings in the appearance of LID, systems of continuous delivery of dopaminergic drugs, such as the continuous intraduodenal infusion of l-DOPA (known as DuoDopa), are less susceptible of inducing dyskinesias (11, 12). In fact, this treatment strategy is adopted in cases of advanced disease, where motor fluctuations are no longer manageable by modifying the regimen of the oral therapy.

## The Serotonergic System in LID: Pre-Clinical Evidence

Although the efficacy of the treatment is partly compromised in advanced stage of disease, as it is in animal models of complete DA denervation, l-DOPA still produces clear motor effects, of which dyskinesias represent an abnormal manifestation; this suggests that other cellular compartments can substitute the lost DA neurons in mediating l-DOPA conversion to DA, and neurotransmitter release. In this context, the serotonergic system has emerged, in recent years, as a key player (1). In fact, serotonin neurons share with the DA ones, the same enzymatic machinery required to convert l-DOPA to DA and mediate vesicular storage, the aromatic amino acid decarboxylase, and monoamine vesicular transporter, respectively. In agreement, early studies have demonstrated the ability of serotonin neurons to store DA after exogenous administration of l-DOPA (13, 14). However, serotonin neurons lack a feedback control mechanism able to fine-tune the synaptic levels of DA. As consequence, l-DOPA-derived DA is released in an uncontrolled manner, leading to excessive synaptic DA peaks, and contributing to swings in synaptic DA levels following oral administration of l-DOPA; this will eventually determine pulsatile stimulation of striatal post-synaptic DA receptors, and changes in signaling cascades at striatal neurons (5, 9, 15–18).

Serotonin neurons are supposed to be involved in the releasing of DA also in early stages of disease; however, such contribution may initially be beneficial due to the presence of the spared DA terminals that can buffer serotonin neuron-derived DA and avoid excessive DA receptor stimulation (1). In support of this view, it has recently been shown that a 30% reduction of striatal l-DOPA-derived DA release is induced upon removal of serotonin nerve fibers in DA neuron-intact rats (19).

Hence, as the DA neuron degeneration progresses, serotonin neurons are expected to contribute more and more to conversion of exogenously administered l-DOPA to DA, eventually producing excessive DA receptor activation. In line with this view, removal of serotonin innervation by 5,7-dihydroxytryptamine (5,7-DHT) administration reduced l-DOPA-derived extracellular DA levels by about 80% in the striatum of complete DA-lesioned rats (20). Most importantly, removal of forebrain serotonin innervation leads to near-complete suppression of LID in l-DOPA-primed parkinsonian rats (15).

The involvement of serotonin neurons in the appearance of LID in animal models has also been demonstrated with a pharmacological approach. In fact, silencing of serotonin neurons can be achieved by targeting the serotonin auto-receptors with selective agonists. Accordingly, several studies have shown a reduction of LID induced by selective 5-HT1 receptor agonists in pre-clinical animal models of PD (15, 16, 21–24). Moreover, co-administration of 5-HT1A and 5-HT1B receptor agonists (8-OHDPAT and CP-94253, respectively) in parkinsonian rats has been demonstrated to produce a synergistic effect on reduction of LID, with complete suppression at doses of the drugs that were ineffective when given individually (15). Most importantly, this result was achieved also in dyskinetic MPTP-treated macaques (24). In line with this hypothesis, reduction of extracellular DA levels was found to account for the potent anti-dyskinetic effect of 5-HT1A and 5-HT1B receptor agonists on LID in a following rat microdialysis study (9).

Interestingly, similar results were also obtained with mixed 5-HT1A/1B receptor agonists, such as eltoprazine and anpirtoline in both rats and macaques, albeit a partial worsening of the therapeutic effect of l-DOPA was seen at effective doses (25, 26). In further support of a pre-synaptic action of 5-HT1A/1B receptor agonists, doses able to fully suppress LID were ineffective against dyskinesia produced by the DA direct agonist apomorphine (26, 27). This is relevant as 5-HT1A and 5-HT1B receptors are also located post-synaptically, and their activation has been shown to reduce striatal glutamate and GABA release, respectively (28, 29), which is known to produce anti-dyskinetic effects; indeed, higher doses of these compounds have been demonstrated to also reduce dyskinesia induced by apomorphine (23).

The involvement of the serotonin neurons in the appearance of LID has also been demonstrated in a rat PET-imaging study; in fact, Nahimi and co-workers have shown that administration of 8-OH-DPAT could reverse l-DOPA-induced decrease of [(11) C]raclopride binding and increase of extracellular DA in 6-OHDA-lesioned rats (30).

The interaction between l-DOPA and serotonin neurons has been shown in another recent study, where administration of the serotonin immediate precursor 5-hydroxy-tryptophan (5-HTP) has produced anti-dyskinetic effect in parkinsonian rats; this effect has been demonstrated to be partly mediated by activation of serotonin auto-receptors and partly by displacement of DA from serotonergic vesicles produced by the exogenous 5-HTP-derived serotonin (31).

Even more striking results were obtained using selective serotonin reuptake inhibitors (SSRIs), such as citalopram or fluoxetine (32, 33), which are known to exert their anti-depressant effect by increasing synaptic serotonin levels. In these studies, the authors observed complete suppression of LID at relatively low doses of drugs, while a 5-HT1 receptor antagonist appeared to counteract this effect, suggesting that increased activation of serotonin auto-receptors is involved in the mechanisms of the anti-dyskinetic effect of SSRIs in parkinsonian rats.

It should be noted that SSRIs and 5-HTP were found to produce clear anti-dyskinetic effect without compromising the therapeutic efficacy of l-DOPA in specific motor tasks. This is highly relevant as it may suggest that worsening of the efficacy of l-DOPA seen with selective 5-HT1 receptor agonists in parkinsonian rats may be due to a transient reduction of the serotonergic tone. In fact, selective 5-HT1 receptor agonists are expected to reduce both DA and serotonin release from serotonin neurons; by contrast, 5-HTP and SSRIs are likely to reduce DA release without dampening synaptic serotonin levels. This is mostly important when thinking that many advanced PD patients also suffer for symptoms of depression, and administration of selective 5-HT1 receptor agonists may exacerbate this complication.

It should be noted that dampening of serotonin neuron release by 5-HT1 receptor agonists did not only reduce LID, but it has also been shown to prevent induction of post-synaptic alterations at striatal neurons, such as increased expression of FosB and altered synaptic NMDA receptor subunits distribution (24). This confirms that false transmitter release of DA from serotonin neurons plays a key role in driving the maladaptive alterations that associate with dyskinesia.

## The Serotonergic System in LID: Clinical Evidence

Whereas an overwhelming body of evidence proves a major role of the serotonergic system in the appearance of LID in animal model, clinical evidence are still scarce.

A large clinical study was conducted in the past years to investigate the efficacy sarizotan, a partial 5-HT1A receptor agonist, in dyskinetic patients. Despite the promising results obtained in pre-clinical experiments, and in an open-label study, the double-blind investigation was terminated for lack of efficacy (34, 35). While disappointing, these results may be due to the fact that sarizotan also exerts antagonistic activity at the level of the D2 receptor (36). Moreover, it should be noted that sarizotan acts only on the 5-HT1A receptor, while experimental evidence demonstrated that a potent synergistic effect on suppression of LID is obtained by simultaneous targeting of the 5-HT1A and 5-HT1B auto-receptors (24, 26). Indeed, the mixed 5-HT1A/1B receptor agonist eltoprazine, which we recently proved to be highly potent on suppression of LID in animal models, is currently under clinical investigation in a small group of dyskinetic patients, with encouraging preliminary results (see http://www.psychogenics.com/press2012.html).

Although it does not provide direct evidence for the involvement of the serotonin system in LID, the results of the PET-imaging study performed by de la Fuente-Fernandez et al. (5), support the concept that dyskinesia is associated to dysregualted DA release; in fact, in this study, dyskinetic patient showed higher synaptic DA levels 1 h after l-DOPA administration compared to stable responders.

In the same line, a key study has recently been performed by Politis and co-workers (37). First, these authors showed that PD patients with LID had relative preservation of serotonergic terminals compared to patients with stable response to l-DOPA, which correlated with the severity of LID, in agreement with a previous post-mortem investigation (38). Moreover, in patients with LID the same l-DOPA dose induced significant higher striatal synaptic DA levels than in non-dyskinetic patients, as already seen by de la Fuente-Fernandez et al (5). Most importantly, the partial 5-HT1A receptor agonist buspirone, administered orally 15 min before l-DOPA, significantly reduced the l-DOPA-evoked rises in striatal synaptic DA release and attenuated LID (37). Whereas a previous report has shown a partial reduction of LID following buspirone administration (39), the study of Politis and colleagues provides the first direct evidence that such reduction is linked to decreased synaptic DA levels.

Whereas these results are extremely encouraging, a major concern that emerged from animal studies is the preservation of the l-DOPA therapeutic effect following dampening of serotonin neurons activity. Thus, larger clinical studies should be performed to address whether the therapeutic window is sufficient to take full advantage from this approach to counteract LID in parkinsonian patients, or to identify the subset of patients that are more likely to benefit from 5-HT1 receptor agonists.

## Conflict of Interest Statement

The authors declare that the research was conducted in the absence of any commercial or financial relationships that could be construed as a potential conflict of interest.
